# Effects of Moderate Glycemic Control in Type II Diabetes With Insulin on Arterial Blood Gas Parameters Following Coronary Artery Bypass Graft Surgery

**DOI:** 10.5812/cardiovascmed.17857

**Published:** 2014-05-10

**Authors:** Gholamreza Masoumi, Rasoul Frasatkhish, Alireza Jalali, Mohsen Ziyaeifard, Ali Sadeghpour-Tabae, Mojtaba Mansouri

**Affiliations:** 1 Department of Anesthesiology, Shahid Chamran Hospital, Isfahan University of Medical Sciences, Isfahan, IR Iran; 2 Rajaie Cardiovascular Medical and Research Center, Iran University of Medical Sciences, Tehran, IR Iran; 3 Department of Anesthesiology, Baqiyatallah University of Medical Sciences, Tehran, IR Iran

**Keywords:** Diabetes Mellitus, Coronary Artery Bypass, Diabetes Complications, Blood Glucose

## Abstract

**Background:**

Coronary artery bypass grafting (CABG) is one of the common surgeries in patients with coronary artery disease (CAD). It is more probable for patients with diabetes to undergo surgeries due to CAD and they have a higher mortality rate compared to the others

**Objectives:**

The aim of the present study was to assess the effects of insulin infusion therapy on blood gas parameters in two groups of patients, eligible for CABG, defined as A: well controlled diabetes (HbA1C < 7%) and B: non-diabetic patients.

**Patients and Methods:**

We followed two groups of patients, defined as patients with and without diabetes who were candidates for CABG, between March 2010 and March 2012. Patients with diabetes underwent moderate or semi-tight glycemic control, using continuous intravenous insulin infusion.

**Results:**

There were 13 male and 18 female subjects in the on-diabetic group and 11 male and 7 female patients in the controlled diabetic group. There was no significant difference between the studied participants regarding age, cardiac ejection fraction, blood pH and PO_2_ and PCO_2_ levels.

**Conclusions:**

CABG surgery adversely affects arterial blood gas (ABG) determinations. On the other hand, findings showed there is no significant difference in the ABG parameters between patients with well controlled diabetes and the ones without.

## 1. Background

Diabetes mellitus (DM) is the most common metabolic disease, having high rates of mortality and morbidity (1). Since prevalence of DM is increasing, surgeries due to its complications are also increasing (2). Cardiovascular disorders are the most important causes of mortality in patients with diabetes and is three to seven times more common in these patients compared to the general population (3).

As coronary artery surgeries are of the most common surgeries to improve quality of life and surveillance in patients with diabetes, attention to the complications of these surgeries is necessary (4). There are some challenges about blood gas control before, during and after coronary artery bypass grafting (CABG) surgeries and effects of glucose level on outcomes of surgeries (5). It is also clear that perioperative hyperglycemia is harmful for patients undergoing cardiac surgery (6). Although benefits of blood glucose control are clear, there are some challenges on the optimum level of blood glucose in these patients (7, 8). Some studies showed no significant difference in pre and postoperative care of CABG patients, between aggressive glycemic control (90-120 mg/dL) and moderate glycemic control (120-180 mg/dL) (9, 10).

## 2. Objectives

The aim of the present study was to assess the effects of diabetes on ABG parameters in two groups of patients with CAD, undergoing CABG, defined as A: well controlled (HbA1C < 7%) diabetes and B: non-diabetic patients.

## 3. Patients and Methods

We followed 49 patients with CAD, undergoing CABG, including 18 patients with controlled type II diabetes as cases and 31 age and BMI matched non-diabetic patients as controls. Patients and controls were randomly selected from the CABG candidates, at Shahid Chamran Hospital, Isfahan, Iran, March 2010 – March 2012. Patients, candidate for CABG were diagnosed according to the criteria of the American college of cardiology (11). CABG, was performed according to the guidelines of American College of Cardiology (12). Diabetes was diagnosed according to the criteria of American Diabetes Association (13). Patients' demographic and anthropometric data were gathered, using a questionnaire. Exclusion criteria were history of heart valve surgery, non-elective CABG, liver or kidney diseases and physical status classification IV and more, according to the American Society of Anesthesiologists (ASA). All participants' HbA1C levels were recorded, two days before the CABG. All participants were fasting for 12 hours before the operation. In the operation room, pulse monitoring, invasive blood pressure monitoring, ECG and capnography monitoring were used and balanced anesthesia with sufentanil, midazolam, Cisatracurium and propofol was used for all participants as mentioned in our previous study (14). Moderate glycemic control (90 - 150 mg/dL) with continuous intravenous insulin solution infusion (50 units of regular insulin in 500 mL of 5% dextrose water, 1 - 5 unit/h) started at the beginning of anesthesia induction and continued until ICU discharge. In addition to blood glucose, ABG control and pulse oxymetry were performed for all participants, before, during and after anesthesia. All patients signed informed written consents before enrollment. The study was conducted in accordance to the Helsinki Declaration and the study protocol was approved by the Ethics Committee at Isfahan University of Medical Sciences (research project number: 36189).

### 3.1. Data Analysis

Independent sample t-test and Mann Whitney U-test were used for data analysis. Data are expressed as mean ± standard deviation (SD). All statistical analyses were performed by SPSS software (version 15; SPSS, Inc., Chicago, IL, USA). P value less than 0.05 was considered statistically significant.

## 4. Results

There were 13 male and 18 female subjects in the healthy group and 11 male and seven female patients in the study group. There was no significant difference between the studied subjects regarding age and arterial blood PH, HCO_3_, PO_2_ and PCO_2_ levels in various intervals (Table 1).

**Table 1.** Comparison of Measured Variables Between Patients, With and Without Diabetes, Undergoing Coronary Artery Bypass Grafting ^a^ , ^b^

	Controlled Diabetic	Nondiabetic (n=31)
**Age, y **	61.4 ± 1.3	59.5 ± 1.5
**HbA1c, % **	6.6 ± 0.9	5.5 ± 0.8
**Arterial PH**		
Before anesthesia	7.40 ± 0.014	7.43 ± 0.005
After anesthesia	7.43 ± 0.011	7.46 ± 0.007
During pump	7.34 ± 0.010	7.36 ± 0.009
During warming	7.40 ± 0.018	7.39 ± 0.01
Off pump	7.36 ± 0.019	7.36 ± 0.011
ICU first 6 hours	7.36 ± 0.011	7.36 ± 0.008
24 Hours after ICU admission	7.40 ± 0.011	7.39 ± 0.008
Discharge	7.41 ± 0.013	7.34 ± 0.078
**Arterial PO2, mmHg**		
Before anesthesia	62.37 ± 2.47	60.65 ± 2.25
After anesthesia	233.50 ± 15.69	237.08 ± 11.46
During pump	340.61 ± 24.13	354.94 ± 11.91
During warming	266.05 ± 23.15	252.54 ± 13.61
Off pump	151.05 ± 17.423	155.66 ± 20.05
ICU first 6 hours	100.33 ± 7.06	101.21 ± 6.05
24 Hours after ICU admission	76.94 ± 6.426	77.23 ± 2.57
Discharge	70.60 ± 3.70	76.35 ± 3.13
**Arterial PCO**_**2**_ **, mmHg**		
Before anesthesia	33.37 ± 1.028	33.18 ± 0.84
After anesthesia	28.88 ± 1.11	27.52 ± 0.77
Pump	32.50 ± 1.37	32.23 ± 1.38
During warming	30.50 ± 1.44	33.93 ± 1.15
Off pump	31.11 ± 1.48	32.24 ± 1.16
ICU first 6 hours	38.00 ± 3.94	37.85 ± 2.61
24 Hours after ICU admission	39.00 ± 3.80	36.61 ± 1.95
Discharge	39.00 ± 4.41	35.73 ± 0.998
**Blood Sugar (surgery day), mg/dL**	138.11 ± 9.54	161.41 ± 9.351

^a^ Data are presented as Mean ± SD.

^b^all the comparisons were not significant (P value >0.05)

## 5. Discussion

CABG surgery adversely affects ABG determinations. The purpose of this study was to assess the serial changes in ABGs, following bypass surgery and also to identify factors that may influence these changes in patients with type II diabetes. Here we studied the effects of moderate glycemic control (blood sugar: 90 - 150 mg/dL), after CABG, on blood gases in 18 patients with type II diabetes and 31 non diabetic controls, using continuous insulin infusion. Our finding showed that the mean ICU length of stay was not significantly different and three days in both groups, (P = 0.811).

This is the first report studying ABG levels, postoperatively in patients with type II diabetes, undergoing CABG. In consistent with our findings, Bucerius et al. and Paun et al. in different studies mentioned that there was no significant difference in the blood gas parameters between patients with and without diabetes, although ICU stay was longer in patients with diabetes (15, 16).

The principal limitation of the present study was its short term follow-up duration. The findings of the current study emphasize on the fact that moderate glycemic control (BS: 90 – 150 mg/dL) cause no statistically significant difference on blood gas and PH levels, in controlled patients with diabetes, compared to non-diabetic patients. The findings of the current study pave the way for future prospective studies with a larger sample size.

## References

[1] Hirsch IB, McGill JB, Cryer PE, White PF (1991). Perioperative management of surgical patients with diabetes mellitus.. Anesthesiology..

[2] Haneya A, Puehler T, Philipp A, Diez C, Ried M, Kobuch R (2011). Coronary artery bypass grafting in patients with type 2 diabetes mellitus: a comparison between minimized and conventional extracorporeal circulation.. ASAIO J..

[3] Ji Q, Mei Y, Wang X, Feng J, Cai J, Sun Y (2010). Impact of diabetes mellitus on patients over 70 years of age undergoing coronary artery bypass grafting.. Heart Lung..

[4] Jones KW, Cain AS, Mitchell JH, Millar RC, Rimmasch HL, French TK (2008). Hyperglycemia predicts mortality after CABG: postoperative hyperglycemia predicts dramatic increases in mortality after coronary artery bypass graft surgery.. J Diabetes Complications..

[5] Martins SK, Santos MA, Tirado FH, Martins FC, Jr., Malat HF, Jatene AD (2007). Coronary artery bypass grafting using both internal mammary arteries in patients with diabetes mellitus.. Rev Bras Cir Cardiovasc..

[6] Giakoumidakis K, Nenekidis I, Brokalaki H (2012). The correlation between peri-operative hyperglycemia and mortality in cardiac surgery patients: a systematic review.. Eur J Cardiovasc Nurs..

[7] Carson J, Scholz PM, Chen AY, Peterson ED, Gold J, Schneider SH (2002). Diabetes mellitus increases short-term mortality and morbidity in patients undergoing coronary artery bypass graft surgery.. Journal of the American College of Cardiology..

[8] Fujii T, Watanabe Y, Shiono N, Kawasaki M, Yokomuro H, Ozawa T (2007). Usefulness of perioperative blood glucose control in patients undergoing off-pump coronary artery bypass grafting.. Gen Thorac Cardiovasc Surg..

[9] Desai SP, Henry LL, Holmes SD, Hunt SL, Martin CT, Hebsur S (2012). Strict versus liberal target range for perioperative glucose in patients undergoing coronary artery bypass grafting: a prospective randomized controlled trial.. J Thorac Cardiovasc Surg..

[10] Lazar HL, McDonnell MM, Chipkin S, Fitzgerald C, Bliss C, Cabral H (2011). Effects of aggressive versus moderate glycemic control on clinical outcomes in diabetic coronary artery bypass graft patients.. Ann Surg..

[11] Pollack CV, Jr, Braunwald E (2008). 2007 update to the ACC/AHA guidelines for the management of patients with unstable angina and non-ST-segment elevation myocardial infarction: implications for emergency department practice.. Ann Emerg Med..

[12] Terkelsen CJ, Sorensen JT, Nielsen TT (2008). Is there any time left for primary percutaneous coronary intervention according to the 2007 updated American College of Cardiology/American Heart Association ST-segment elevation myocardial infarction guidelines and the D2B alliance?. J Am Coll Cardiol..

[13] Nerriere E, Vercambre MN, Gilbert F, Kovess-Masfety V (2009). Voice disorders and mental health in teachers: a cross-sectional nationwide study.. BMC Public Health..

[14] Masoumi G, Pour EH, Sadeghpour A, Ziayeefard M, Alavi M, Anbardan SJ (2012). Effect of different dosages of nitroglycerin infusion on arterial blood gas tensions in patients undergoing on- pump coronary artery bypass graft surgery.. J Res Med Sci..

[15] Bucerius J, Gummert JF, Walther T, Doll N, Falk V, Onnasch JF (2003). Impact of diabetes mellitus on cardiac surgery outcome.. Thorac Cardiovasc Surg..

[16] Paun BC, Cassie S, MacLean AR, Dixon E, Buie WD (2010). Postoperative complications following surgery for rectal cancer.. Ann Surg..

